# *MhCLC-c1*, a Cl channel c homolog from *Malus hupehensis*, alleviates NaCl-induced cell death by inhibiting intracellular Cl^–^ accumulation

**DOI:** 10.1186/s12870-023-04270-3

**Published:** 2023-06-08

**Authors:** Jianfei Song, Mengyuan Han, Xiaoyue Zhu, Huan Li, Yuansheng Ning, Weiwei Zhang, Hongqiang Yang

**Affiliations:** College of Horticulture Science and Engineering, State Key Laboratory of Crop Biology, Shandong Agricultural University, Tai’an, China

**Keywords:** MhCLC-c1, Cl^–^ channel protein, NaCl, Cell death, Cl^–^ accumulation, *Malus hupehensis*

## Abstract

**Background:**

Overaccumulation of chloride (Cl) when plants suffer NaCl causes cell damage and death, and is regulated by Cl^**–**^ channel protein (CLC). Apple roots are very sensitive to Cl^**–**^, but information associated with CLC is limited in apple crop that widely cultivated in the world.

**Results:**

We identified 9 *CLCs* from the apple genome and divided them into two subclasses. Among them, *MdCLC-c1* promoter contained the largest number of *cis-acting* elements associated with NaCl stress, and only the MdCLC-c1, MdCLC-d, and MdCLC-g were predicted that may be Cl^–^ antiporters or channels. Expression analysis of *MdCLCs* homologs in the roots of *Malus hupehensis* showed that most of the *MhCLCs* expression were response to NaCl stress, especially *MhCLC-c1* expression was upregulated continuously and rapidly expressed during NaCl treatment. Therefore, we isolated MhCLC-c1 and observed it was a plasma membrane-localized protein. The *MhCLC-c1* suppression significantly increased sensitivity, reactive oxygen species content, and cell death of apple calli; while *MhCLC-c1* overexpression decreased sensitivity, reactive oxygen species content, and cell death of apple calli and *Arabidopsis* by inhibiting intracellular Cl^–^ accumulation under NaCl stress.

**Conclusions:**

The study selected and isolated a *CLC-c* gene *MhCLC-c1* from *Malus hupehensis* based on identification of *CLCs* gene family in apple, and their homologs *MhCLCs* expression patterns during NaCl treatments, revealing that *MhCLC-c1* alleviates NaCl-induced cell death by inhibiting intracellular Cl^–^ accumulation. Our findings confer the comprehensive and in-depth upstanding of the mechanism that plants resist salt stress, and might also confer genetic improvement of salt tolerance in horticultural crops and the development and utilization of saline–alkali land.

**Supplementary Information:**

The online version contains supplementary material available at 10.1186/s12870-023-04270-3.

## Background

Excess NaCl in soil severely affects crop growth and yield [1]. Chloride (Cl) is a micronutrient for plants, the healthy growth of plants is satisfied by 50–100 μM Cl^–^ only [2, 3]. Although the Cl is thought that may be a macronutrient for plants in some studies, its application concentration is only 1–5 mM [4]. However, the concentration of NaCl solution used for stress treatment is generally 100–200 mM, in which the concentration of Cl^–^ is 20–2000-fold higher than the concentration required for healthy plant growth [5, 6]. When plants suffer NaCl stress, overaccumulation of Cl^–^ in plant cell disturbs nutrient absorption, affects the activity of cytosolic enzymes, and induces the generation of excess reactive oxygen species (ROS), which causes irreversible damage, e.g., cell senescence and death for plants [7, 8]. Moreover, some crops are more sensitivity to Cl^–^, such as citrus [9], apple [10], grape [11], persimmon [12], soybean [13], and tobacco [14]. Moreover, the accumulation of Cl^–^ only in the range of 0.1–5.0 mg·g^–1^ DW can meet the healthy growth of Cl^–^-sensitive plants, e.g., citrus, apple, soybean, and strawberry [15]. Our previous data also showed that Cl^–^-stressed *Malus hupehensis* seedlings generate more higher malondialdehyde (MDA) and ROS than that of Na^+^-stressed under NaCl stress [10]. However, the attention to Cl^–^ toxicity is far less than Na^+^ toxicity in the studies related to NaCl stress [5, 16].

Cl^–^ channel protein (CLC) are highly associated with Cl^–^ accumulation in plants [17]. CLC proteins usually function through monomers or homodimers form, and each of forms has its unique ion conduction pathway [18]. All CLC proteins of eukaryotes contain a Voltage-gate CLC domain and two hydrophilic-regulated cystathionine β synthase (CBS) domains [18, 19]. CLC family proteins also exist three conserved amino acid regions GxGIPE, GKxGPxxH, and PxxGxLF [20]. The x residue in conserved region GxGIPE (I) determines the ion selective filter. The presence of serine (GSGIPE) determines the specific transportation of Cl^–^; whereas if x residue is proline (GPGIPE), NO_3_^–^ is preferentially transported [20]. The previous studies also showed that the conserved gating glutamate (E) in conserved region GKxGPxxH (II), and the proton glutamate (E) residues in the next fourth residue of the conserved region PxxGxLF (III) were signatures for CLC antiporters [21].

To date, the *CLC* genes have been isolated and identified in multiple plants species. There have seven *CLC* members namely from AtCLC-a to AtCLC-g in *Arabidopsis*, and are divided into two subclasses [22]. From AtCLC-a to AtCLC-d and AtCLC-g proteins homologs with eukaryotic CLCs belong to the subclass I, while AtCLC-e and AtCLC-f proteins closed to prokaryotic CLCs are classified into the subclass II [22, 23]. Among them, AtCLC-c has been widely found that promotes plants tolerance to salt stress by regulating Cl^–^ accumulation. Cl^–^ content is significantly less in the guard cells of *clc-c* mutants. Thus, they proposed that AtCLC-c confers Cl^–^ influx into the vacuole and regulates the stomatal opening [24]. Besides, *clc-c* mutants show hypersensitivity to Cl^–^ with a reduction in the shoot as well as root weight [24], whereas overexpression of *AtCLC-c* and co-overexpression of *AVP1*, *PP2A-C5*, and *AtCLC-c* in *Arabidopsis* greatly increases tolerance to salt and drought stresses [25]. The *CLC* gene family is also reported in the other species e.g., Halophyte *Suaeda altissima* [20], wheat [26], *Nicotiana tabacum* [27], soybean [28], *Punica granatum* [29]. Heterogeneous overexpression of *ZmCLC-d* enhances *Arabidopsis* tolerance to cold, drought and salt stress [30]. Both *GmCLC1* and *GsCLC-c2* in soybean confer Cl^–^/salt tolerance by regulating Cl^–^ transport from the roots to the shoots [28, 31]. However, the *CLC-c* genes functions in plants that resist NaCl stress are still not clear, especially in horticultural crops which are sensitive to Cl^–^.

Apple is an important horticultural economic crop widely cultivated in the world. Cultivated apples are grafts of rootstock and scion in production, and its root system, the most direct and earliest organ to perceive and recognize soil salinity, is provided by rootstock. *Malus hupehensis* Rehd. var. *pingyiensis* Jiang is commonly used as apple rootstock that provides root system for the cultivated apples [32], but is susceptible to NaCl, especially to Cl [10]. In the present study, based the identification and comprehensive-analysis of *CLCs* gene family in apple genome (*Malus domestica*), we isolated a *CLC-c* homolog (MhCLC-c1) that strongly induced by NaCl stress from *M. hupehensis* roots, and further revealed that *MhCLC-c1* alleviates the NaCl-induced cell death by inhibiting intracellular Cl^–^ accumulation. Our findings might confer the comprehensive and in-depth upstanding of the mechanism that plants resist salt stress, and might also confer genetic improvement of salt tolerance in horticultural crops and the development and utilization of saline–alkali land.

## Results

### Identification, evolutionary, and conserved regions analysis and of MdCLCs

**Fig. 1:**
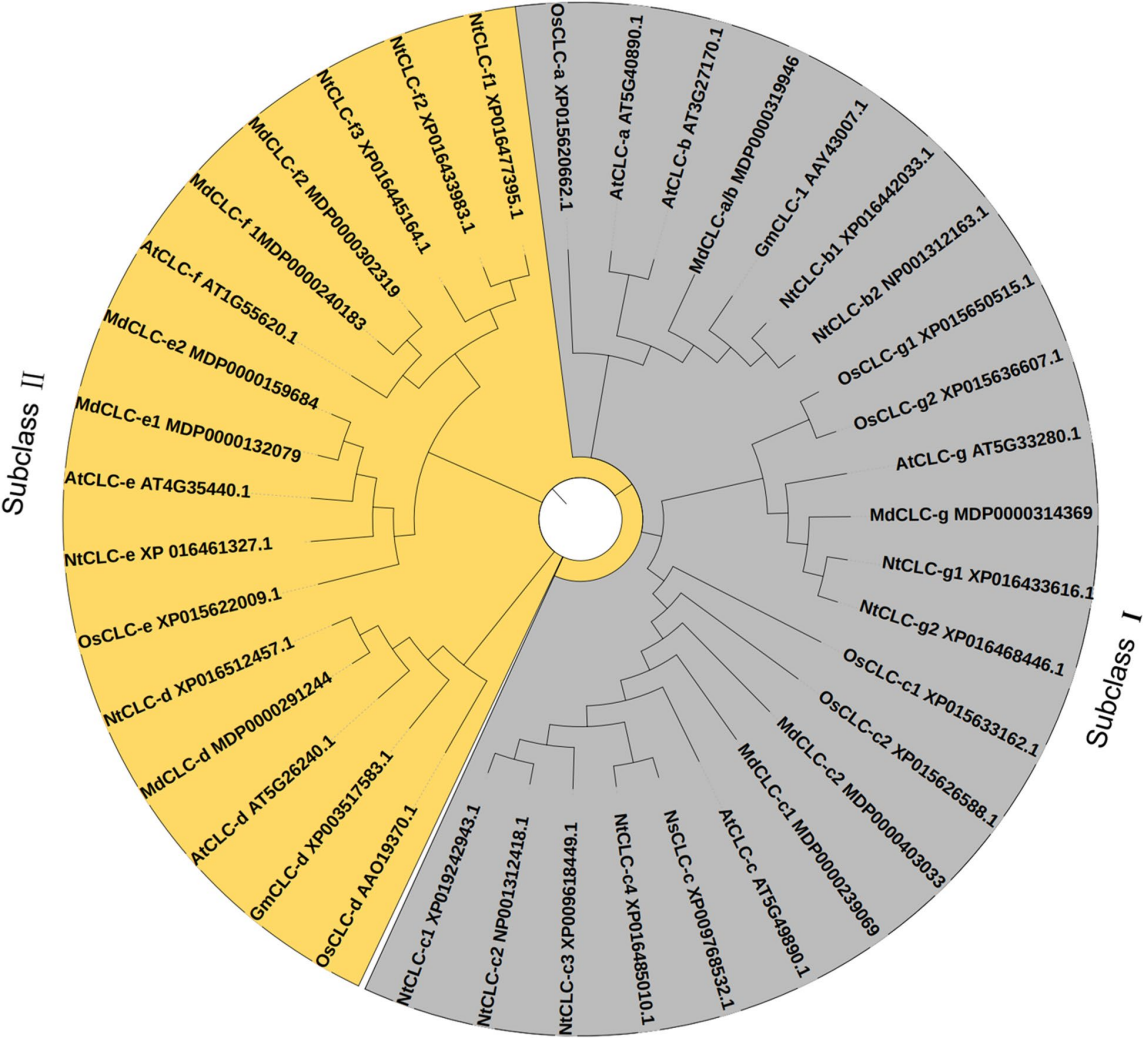
Phylogenetic tree of CLC proteins in plants. The phylogenetic tree was constructed by the neighbor-joining method, including *Malus domestica* (MdCLCs), *Arabidopsis thaliana* (AtCLCs), *Oryza sativa* (OsCLCs), *Nicotiana tabacum* (NtCLCs), *Nicotiana sylvestris* (NsCLCs), and *Glycine max* (GmCLCs). Different colors represent different subclasses

In total, 9 CLCs were identified from apple genome (*Malus domestica*) and renamed based the name of AtCLCs. We further constructed a phylogenetic tree based MdCLCs and their associated homologs from *Arabidopsis thaliana*, *Oryza sativa*, *Nicotiana tabacum*, *Nicotiana sylvestris*, and *Glycine max* using neighbor-joining method. MdCLCs were categorized into two subclasses, of which MdCLC-a/b, MdCLC-c1, MdCLC-c2, MdCLC-d, and MdCLC-g belonged to subclass I, and MdCLC-e1, MdCLC-e2, MdCLC-f1, and MdCLC-f2 belonged to subclass II (Fig. 1). Moreover, only MdCLC-a/b was closely related to AtCLCa and AtCLCb (Fig. 1).

The x residue in conserved region GxGIPE (I) is the signatures for anion selectivity [20]. Among the MdCLCs, only MdCLC-a/b, MdCLC-c1, MdCLC-d, and MdCLC-g contained conserved region GxGIPE (I). The x residue of MdCLC-a/b was proline (GPGIPE), whereas the other three MdCLCs were serine (GSGIPE), indicating that MdCLC-a/b preferentially transported NO_3_^−^, but MdCLC-c1, MdCLC-d, and MdCLC-g specific transported Cl^−^. Moreover, the presence of the conserved gating glutamate (E) in conserved region GKxGPxxH (II) and the proton glutamate (E) residues in the next fourth residue of PxxGxLF (III) serve as signatures for CLC antiporters, however, if any other amino acids exist in these positions, these proteins may exert CLC channels activity [21]. Among the 9 MdCLCs, only MdCLC-a/b, MdCLC-c1, and MdCLC-d had the signatures for CLC antiporters, whereas MdCLC-g, MdCLC-e1, MdCLC-e2, MdCLC-f1, and MdCLC-f2 may exhibit CLC channels activity. Besides, the 9 MdCLCs were predicted to be plasma membrane (PM)-located proteins (Table 1). These results suggest that MdCLC-c1, MdCLC-d, and MdCLC-g may function as CLC antiporters/channels for transporting Cl^−^, involving in the response to NaCl stress.

**Table 1:** Three conserved regions of MdCLCs

Protein name	GxGIPE (I)	GKxGPxxH (II)	PxxGxLF (III)	Predicted function	Anion selectivity	Predicted subcellular localization
MdCLC-a/b	GPGIPE	GKEGPLVH	PVGGVLFALEEVAT	Antiporter	NO_3_^–^	Plasma membrane
MdCLC-c1	GSGIPE	GKEGPMVH	PVGGVLFALEEAAS	Antiporter	Cl^–^	Plasma membrane
MdCLC-c2	/	/	PVGGVLFALEEAAS	—	—	Plasma membrane
MdCLC-d	GSGIPE	GKEGPLVH	PVGGVLFALEEVTS	Antiporter	Cl^–^	Plasma membrane
MdCLC-g	GSGIPE	GKAGPMVH	PVGGVLFAFEEMAS	Channel	Cl^–^	Plasma membrane
MdCLC-e1	/	GPEGPSVE	AVSGCFFAVESVLW	Channel	—	Plasma membrane
MdCLC-e2	/	GPEGPSVE	AVSGCFFAVESVLW	Channel	—	Plasma membrane
MdCLC-f1	/	GPEGPSVD	AVAGCFFAIETVLRP	Channel	—	Plasma membrane
MdCLC-f2	/	GPEGPSVD	AVAGCFFAIETVLRP	Channel	—	Plasma membrane

### Analysis of *MdCLCs* tissue expression profiles and *cis‑acting* elements associated with NaCl stress

**Fig. 2:**
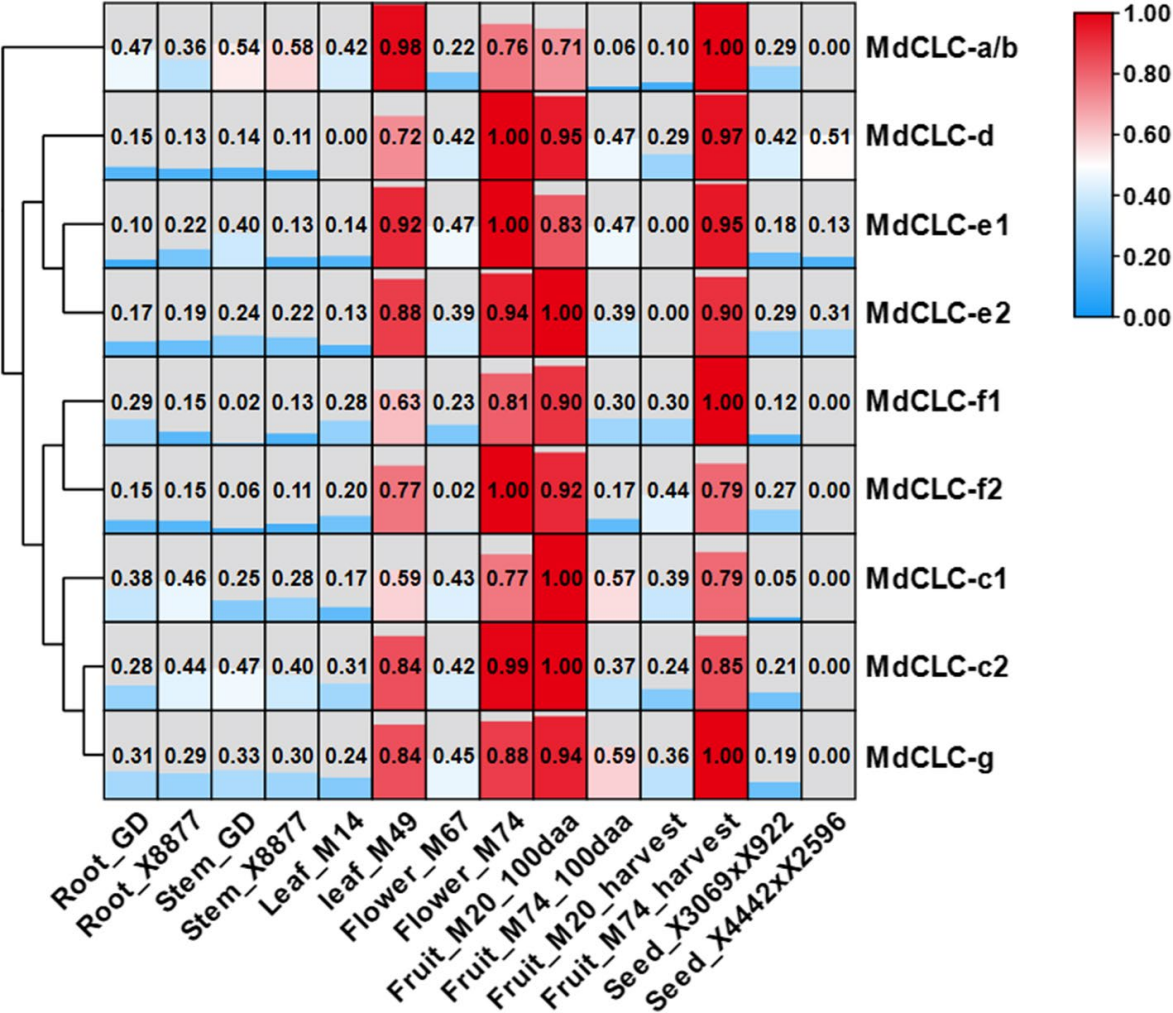
Expression profiles of *MdCLCs* in different tissues of apple. Expression profiles data were downloaded from NCBI-GEO database (GSE42873). Root_GD and Root_X8877 represent the *in virto* root of Golden Delicious and X8877, respectively; Stem_GD and Stem_X8877 represent the fully developed-stem of Golden Delicious and X8877, respectively; Leaf_M14 and Leaf_M49 represent the whole leaf of M14 and M49, respectively; Flower_M67 and Flower_M74 represent the whole flower of M67 and M74, respectively; Fruit_M20_100aa and Fruit_M74_100aa represent the 100 days after anthesis-fruit of M20 and M74, respectively; Fruit_M20_harvest and Fruit_M74_ harvest represent the fruit flesh at harvest of M20 and M74, respectively; Seed_X3069xX922 and represent the dormant seed from cross X3096 and X922; Seed_X4442xX2596 represent the dormant seed from cross X4442 and X2596, respectively. M67, M74, M20, M14, M49, X8877, Golden Delicious, X41002, X4442 and X2596 represent apple cultivars. Every RPKM value of sample was obtained according to the mean of two replication, and the data was standardized and normalized by log2 and zero to one, respectively. The heatmap was constructed using Tbtools

Based the NCBI-GEO database (GSE42873), we further analyzed the tissue expression profiles of *MdCLCs* family. All *MdCLCs* have a higher expression level in leaves, fruits and flowers than the other organs. Compared with the others, *MdCLC-a/b*, *MdCLC-c1, MdCLC-c2,* and *MdCLC-g* had the higher expression level in root, especially *MdCLC-a/b* and *MdCLC-c1* (Fig. 2), implying that *MdCLC-c1* is most likely involved in the response of apple to soil stress. Moreover, *MdCLC-a/b* and *MdCLC-c2* had the higher expression level in stems, while *MdCLC-d* and *MdCLC-e2* had the higher expression level in seeds (Fig. 2).

**Fig. 3:**
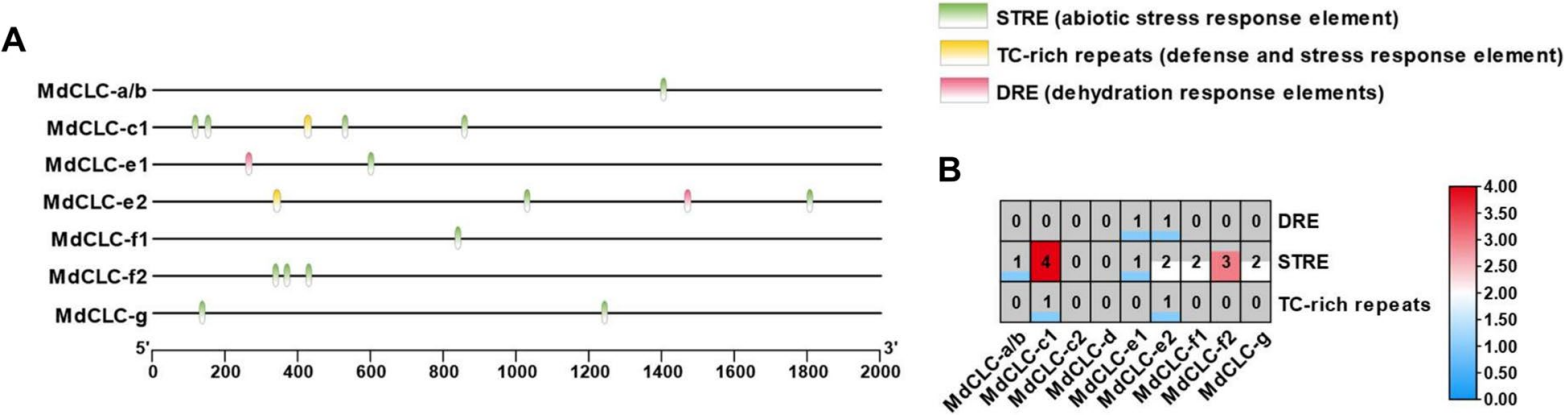
Analysis of *cis-acting* elements associated with NaCl stress in *MdCLCs* promoters. **A** The distribution of *cis-acting* elements associated with NaCl stress in *MdCLCs* promoters. The 2 kb promoters’ sequence from the upstream of translation start site (ATG) were analyzed using plantCARE, and the different color represent different *cis-acting* element. **B** The number of different *cis-acting* elements associated with NaCl stress in the *MdCLCs’* promoters. The word in every box represents the number of each *cis-acting* element

Further the *cis-acting* elements associated with NaCl stress were scanned in promoters of *MdCLCs* (Fig. 3A). Except the promoters of *MdCLC-c2* and *MdCLC-d*, all the other *MdCLCs’* promoters had *cis-acting* elements that responds to NaCl stress, of which *MdCLC-c1* promoter had the largest number NaCl stress response elements (4 STRE and 1 TC-rich repeats) (Fig. 3B). These findings indicate that compared with the other *MdCLCs*, *MdCLC-c1* may be the most possible member which be involved the responses of plants to NaCl stress.

### *MhCLCs* expression patterns in response to NaCl stress

**Fig. 4:**
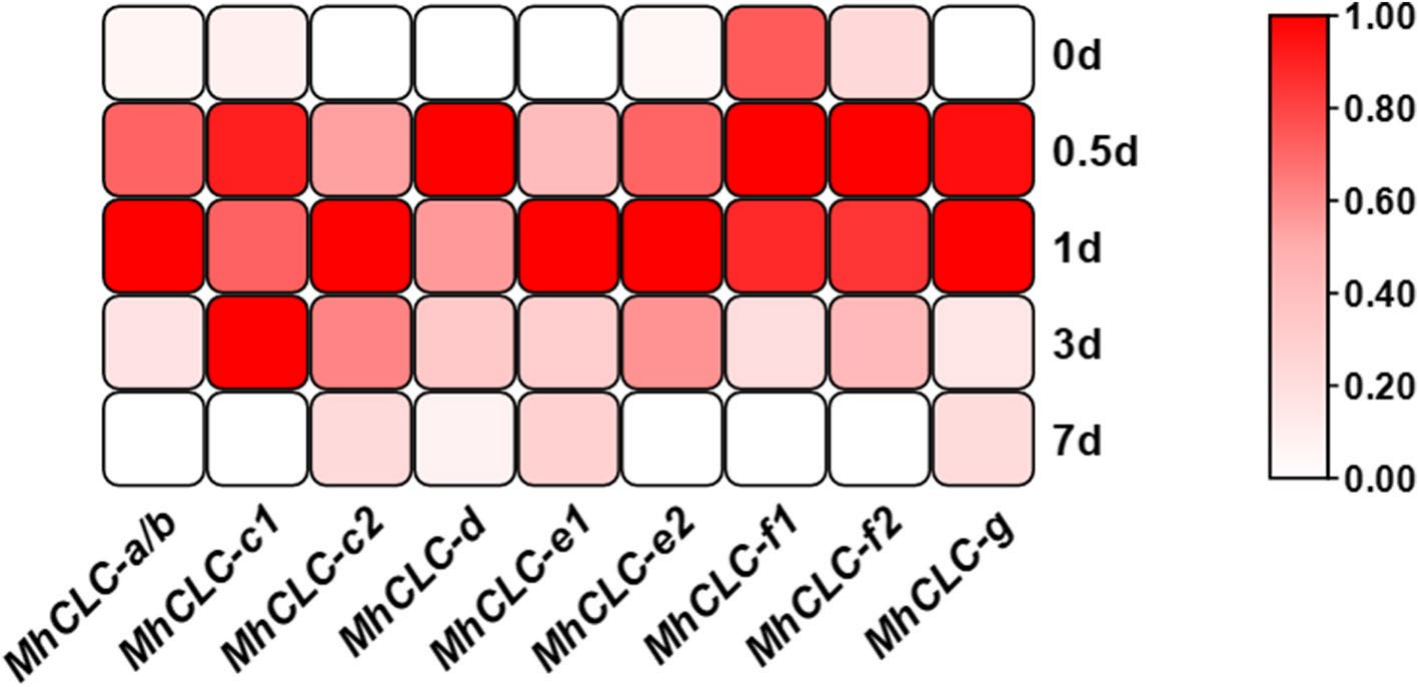
Effect of NaCl treatments on *MhCLCs* expression. The relative expression level of *MhCLCs* after 150 mM NaCl treatment for 0, 0.5, 1, 3, 7 d. The relative expressional levels were calculated by the 2^−ΔΔCT^ method. The data was normalized by zero to one method

Considered that the *MdCLCs* may be involved in the response to NaCl stress implied by the given *cis-acting* elements analysis (Fig. 3), and the excess NaCl in soil is earliest and most directly identified by roots that provided by rootstock in cultivated apple. Thus, we detected the *MdCLCs* homologs *MhCLCs* expression under NaCl treatment in *M. hupehensis* roots, an excellent apple rootstock. Except for *MhCLC-c1*, the other 8 *MdCLCs* expression significantly upregulated within 1 d of NaCl treatment, followed by a return to baseline levels after 3 d of NaCl treatment (Fig. 4). In contrast, *MhCLC-c1* expression remained significantly elevated for d 3 d of NaCl treatment, and then returned to baseline levels after 7 d (Fig. 4). Moreover, *MhCLC-c1*, *MhCLC-d*, *MhCLC-f1*, *MhCLC-f2*, and *MhCLC-g* expression reached peak levels after 0.5 d of NaCl treatment, implying that they may have a rapid response to NaCl stress (Fig. 4). In conclusion, *MhCLC-c1* expression exhibited more unique changes compared to other members of MhCLCs. Therefore, the *MhCLC-c1* was selected for further study.

### Isolation and subcellular localization of MhCLC-c1

**Fig. 5:**
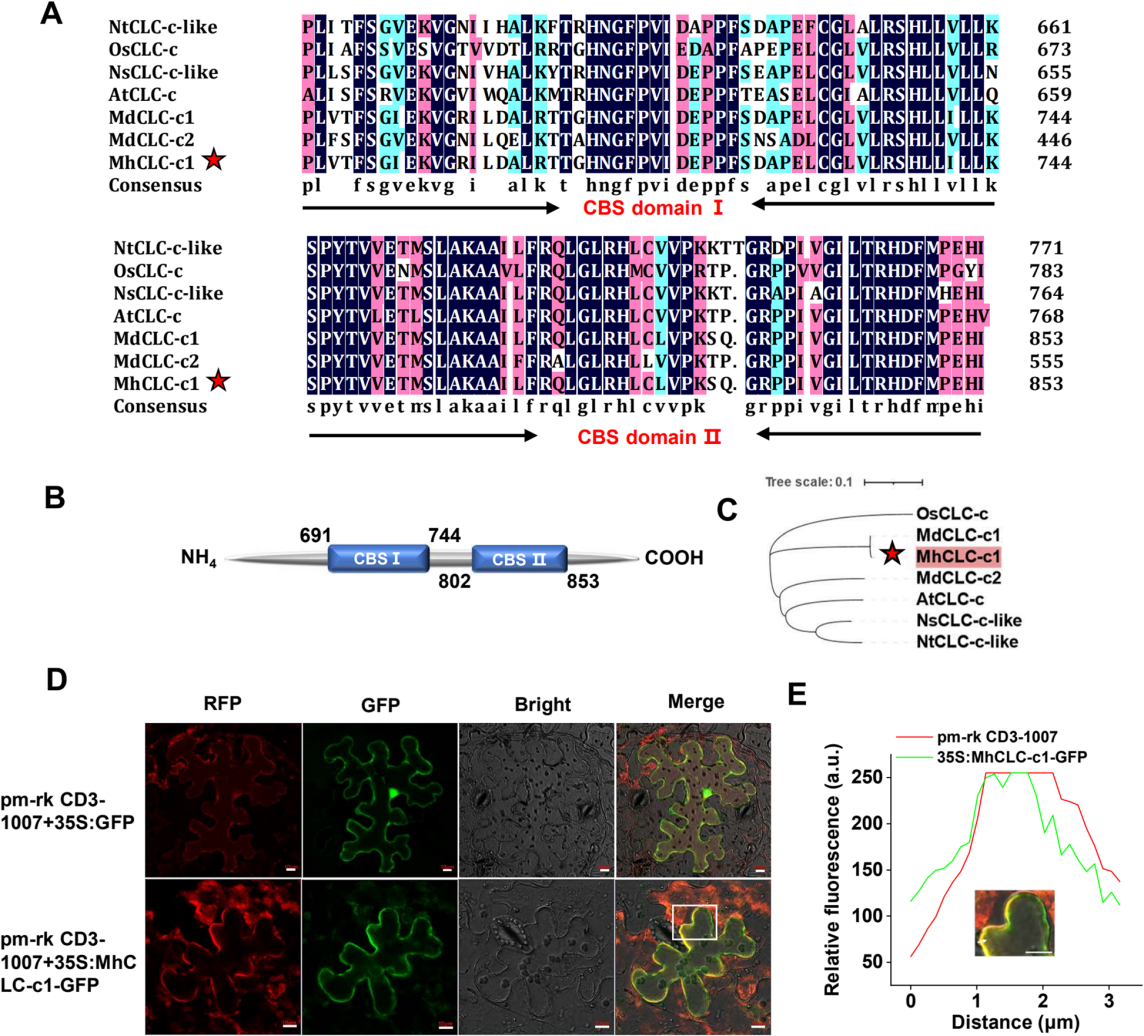
Evolutionary analysis and subcellular location of MhCLC-c1 from *Malus hupehensis*. (**A**) Multiple alignments, (**B**) domain architecture analysis, and (**C**) phylogenetic analysis of MhCLC-c1 and its homologs from tobacco, apple, rice, rose, and *Arabidopsis*. The MhCLC-c1 was pointed using pentagram. **D** Subcellular localization of 35S:GFP and 35S:MhCLC-c1-GFP. A plasma membrane-located protein pm-rk CD3-1007 was transiently co-transformed with 35S:GFP or 35S:MhCLC-c1-GFP in tobacco leaves, which used as the maker of plasma membrane. Scale bar = 10 μm. **E** The intensity profile analysis of RFP (pm-rk CD3-1007) and GFP fluorescence. The measured location was pointed using arrow. Scale bar = 10 μm

Further, we selected the MhCLC-c1 to explore its role in the response to NaCl stress. Based the MdCLC-c1 sequence, we isolated the MhCLC-c1 from *M. hupehensis* roots. Consistent with the CLC-c from *Arabidopsis thaliana*, *Oryza sativa*, *Nicotiana tabacum*, *Nicotiana sylvestris*, and *Glycine max*, MhCLC-c1 also had two CBS domain at sites 691–744 and 802–853 (Fig. 5A, B), and the MhCLC-c1 had the close genetic relationship with the CLC-c1 from the other species, especially MdCLC-c1 (Fig. 5C). Furthermore, the subcellular localization of MhCLC-c1-GFP fusion protein was performed through *Agrobacterium* GV3101 infiltration of tobacco leaves, and observed that the MhCLC-c1 is PM-localized protein (Fig. 5D). In addition, the similar fluorescence intensity curves at the same location of MhCLC-c1-GFP and pm-rk CD3-1007 (a PM-located protein) [33], also supported the result that MhCLC-c1 is PM-localized protein (Fig. 5E). These results imply that MhCLC-c1 may play a role in PM.

### MhCLC-c1 alleviates NaCl-induced cell death

**Fig. 6:**
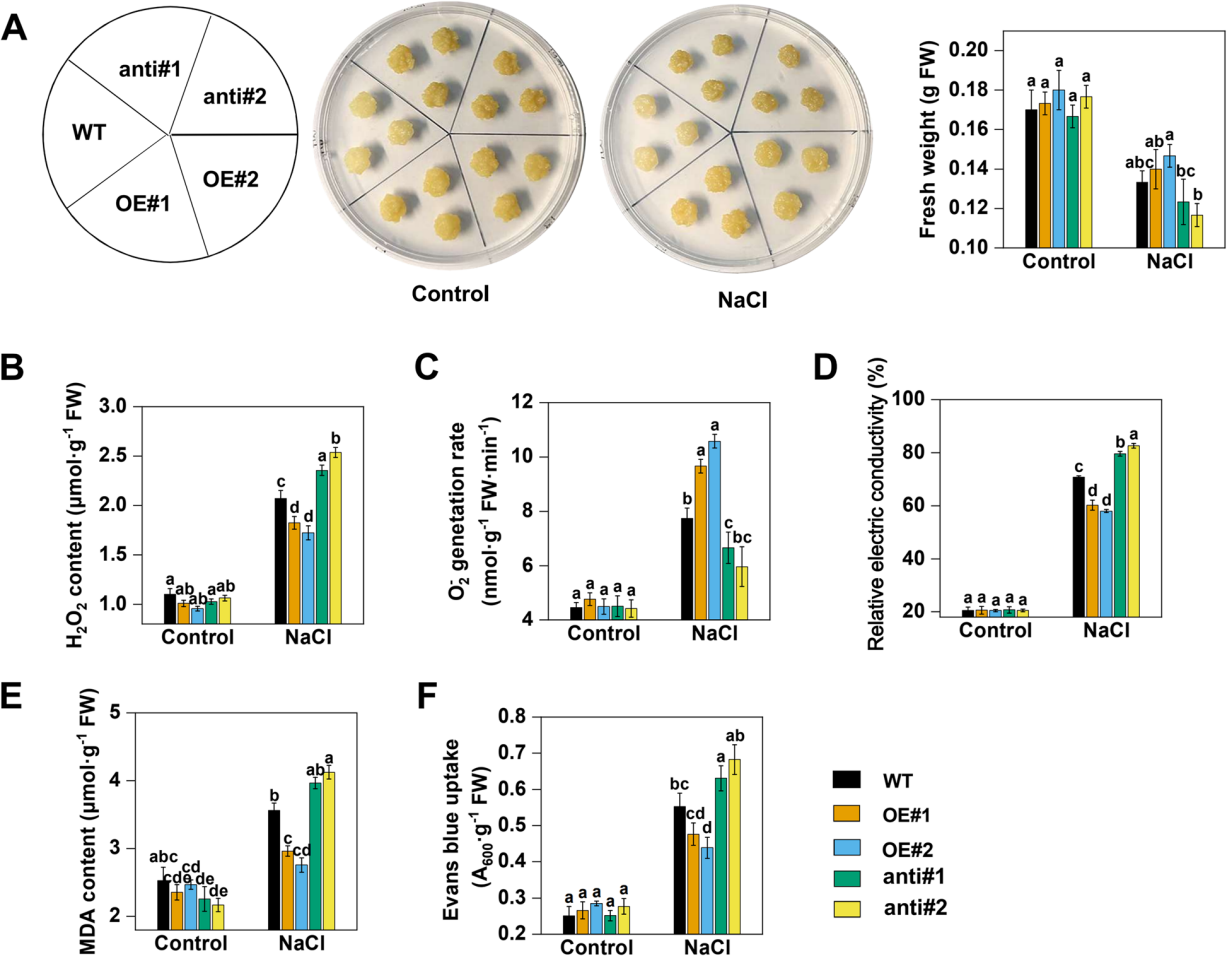
Effect of MhCLC-c1 on cell death under NaCl stress in apple calli. **A** The growth phenotypes and fresh weight of wild-type (WT), *MhCLC-c1* overexpressing (OE#1 and #2), and *MhCLC-c1* suppressing (anti#1 and #2) apple calli. (**B**) H_2_O_2_ content, (**C**) O_2_^–^ generation rate, and (**D**) REC, and (**E**) MDA content of apple calli shown in Fig. 6A. **F** The Evans blue uptake values indicated cell death and. Control: 15-d-old apple calli grown in MS medium for 15 d; NaCl: 15-d-old apple calli grown in MS medium supplementing 200 mM NaCl for 15 d

To determine the role of MhCLC-c1 in NaCl-induced cell death, the apple calli overexpressing *MhCLC-c1* (OE#1 and OE#2) and that suppressing *MhCLC-c1* (anti#1 and anti#2) were generated (Figure S1A). Under normal condition, the growth and fresh weight (FW) of five apple calli had no significant difference (Fig. 6A). However, the OE had higher FW than that of wild-type (WT) and anti apple calli, while the anti had lower FW than that of WT after NaCl stress (Fig. 6A). Moreover, the apple calli overexpressing *MhCLC-c1* exhibited lower hydrogen peroxide (H_2_O_2_) content, superoxide anion (O_2_^–^) generation rate, relative electric conductivity (REC), and MDA content, but that suppressing *MhCLC-c1* had higher H_2_O_2_ content, O_2_^–^ generation rate, REC, and MDA content under NaCl stress (Fig. 6B–E). Further, we used an Evans blue (EB) dye to evaluate cell death and observed that the OE exhibited lower EB dying degree than the others, but the anti had higher EB dying degree than WT (Fig. 6F). These findings indicate that MhCLC-c1 alleviates apple calli sensitivity to NaCl stress and NaCl-induced cell death.

**Fig. 7:**
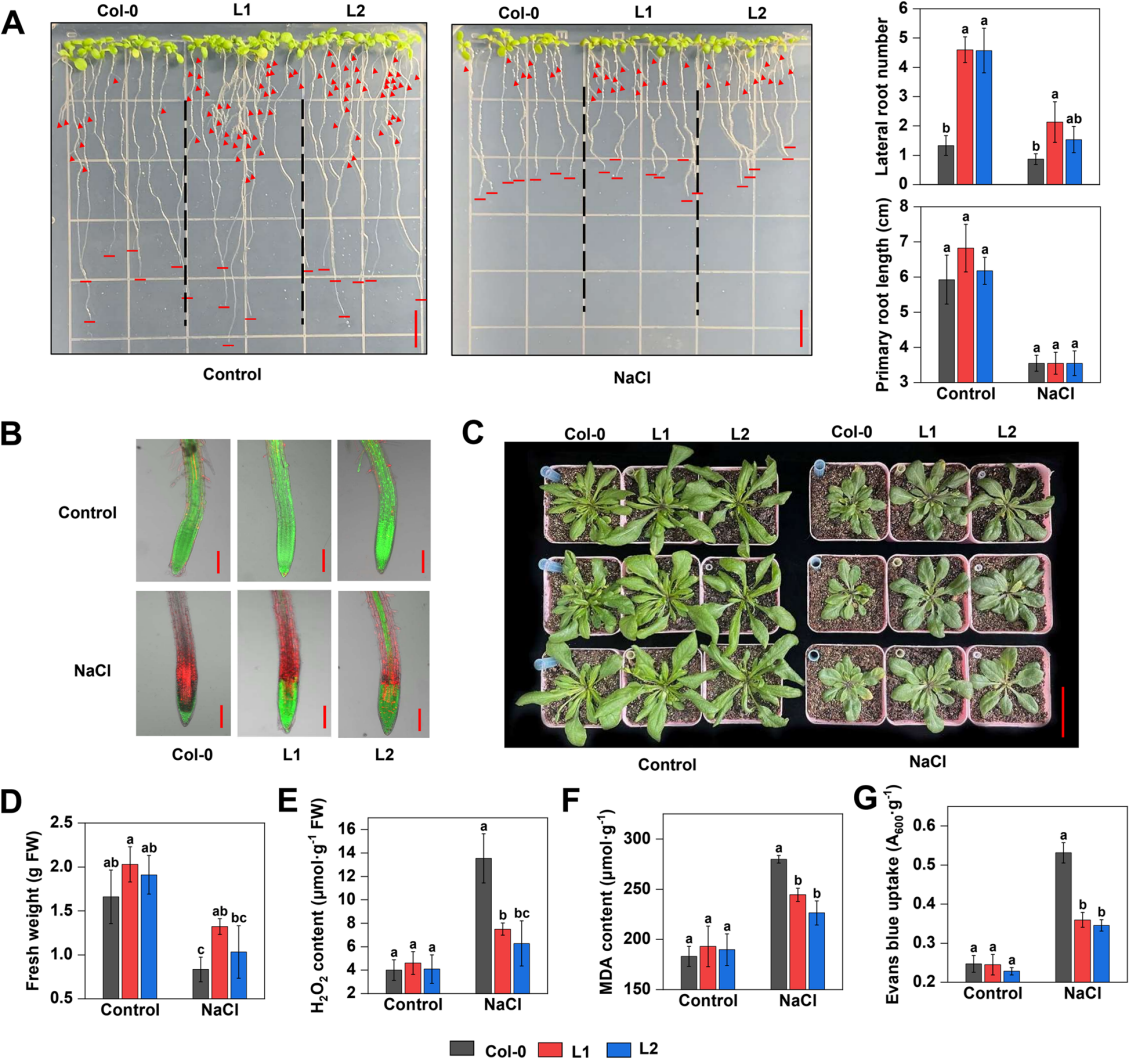
Effect of MhCLC-c1 on cell death under NaCl stress in *Arabidopsis*. **A** The roots growth phenotypes, lateral roots (LR) number, and primary root length of wild-type (Col-0) and *MhCLC-c1* overexpressing *Arabidopsis* (L1 and L2). Control: the 4-d-old *Arabidopsis* cultivated in ½ MS medium for 5 d; NaCl: the 4-d-old *Arabidopsis* cultivated in ½ MS medium supplementing 100 mM NaCl for 5 d. Scale bar = 1 cm. **B** The FDA/PI double staining of LR shown in Fig. 7A. Scale bar = 100 μm. **C** The growth phenotypes of Col-0, L1, and L2 *Arabidopsis* on soil. Control: the 10-d-old *Arabidopsis* was watered using ½ Hoagland’s nutrient solution once every two days; NaCl: the 10-d-old *Arabidopsis* was watered using ½ Hoagland’s nutrient solution containing 200 mM NaCl once every two days. Scale bar = 5 cm. (**D**) The fresh weight, (**E**) H_2_O_2_ content, (**F**) MDA content, and (**G**) Evans blue uptake values indicated cell death of *Arabidopsis* shown in Fig. 7C. Bar represents mean ± SD and different letters above a bar represent a significant difference (*p* < 0.05)

Moreover, we also generated the *Arabidopsis* overexpressing *MhCLC-c1* (L1 and L2) (Figure S1B). The primary root length between Col-0, L1, and L2 had no significant difference (Fig. 7A). However, the L1 and L2 had more lateral roots (LR) than Col-0 whether normal or NaCl stress conditions (Fig. 7A). Further, an FDA/PI double staining showed that the L1 and L2 exhibited more FDA (green) fluorescence and less PI (red) fluorescence under NaCl stress, which indicated that the L1 and L2 had higher cell activity than Col-0 (Fig. 7B). Moreover, the FW between the three *Arabidopsis* plants had no significant difference when they were normally cultivated on soil (Fig. 7C). However, the L1 and L2 showed higher FW, but lower H_2_O_2_ content, MDA content, and cell death (indicated by EB uptake values) lower than Col-0 when watered by NaCl solution (Fig. 7C–F). These results suggest that MhCLC-c1 decreases *Arabidopsis* sensitivity to NaCl stress and NaCl-induced cell death.

### MhCLC-c1 inhibits intracellular Cl^–^ accumulation under NaCl stress

**Fig. 8:**
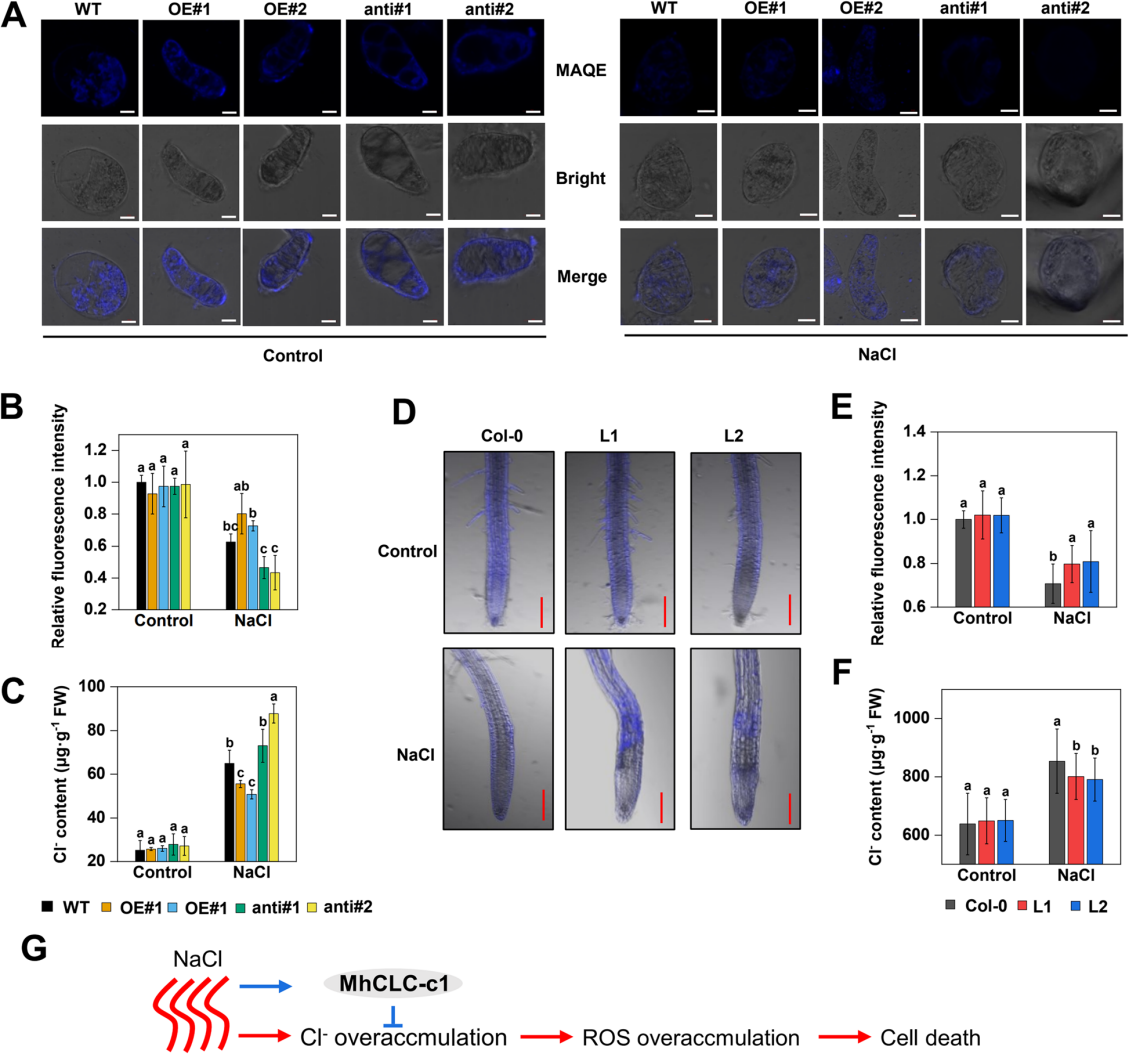
Effect and the work model of MhCLC-c1 on Cl^–^ content under NaCl stress. **A** Cl^–^ accumulation phenotypes in apple calli shown in Fig. 6A. A Cl^–^ probe MAQE was used to measure Cl^–^ accumulation, and its fluorescence intensity decreases with the increase of Cl^–^ content. Scale bar = 10 μm. **B** The relative fluorescence intensity of apple calli shown in Fig. 8A. **C** The quantitative of Cl^–^ content shown in Fig. 6A. **D** Cl^–^ accumulation phenotypes in *Arabidopsis* roots shown in Fig. 7A. Scale bar = 100 μm. **E** The relative fluorescence intensity of *Arabidopsis* shown in Fig. 7D. **F** The Cl^–^ content of whole *Arabidopsis* plants shown in Fig. 7C. **G** A work model for MhCLC-c1 alleviates NaCl-induced cell death through inhibiting intracellular Cl^–^ accumulation. NaCl stress induced the expression of *MhCLC-c1*, and the *MhCLC-c1* alleviates the Cl^–^ overaccumulation caused by NaCl stress, thus the ROS overaccumulation and cell death caused by NaCl was alleviated. Bar represents mean ± SD and different letters above a bar represent a significant difference (*p* < 0.05)

Function prediction showed that MdCLC-c1 may be a selective transporter for Cl^–^ (Table 1). Therefore, further the intracellular Cl^–^ content was measured using a fluorescent probe N-(Ethoxycarbonylmethyl)-6-Methoxyquinolinium Bromide (MQAE) whose fluorescence intensity decreases with increasing Cl^–^ content. The MQAE fluorescence intensity of five apple calli had no significant difference under normal conditions, however, the OE exhibited higher fluorescence intensity than the others, but the anti had lower fluorescence intensity than WT under NaCl stress (Fig. 8A, B). And the measurement of Cl^–^ content also supported the fluorescence intensity results (Fig. 8C). Besides, the Cl^–^ content in *Arabidopsis* was also determined. Compared to the Col-0, the higher MQAE fluorescence intensity and lower Cl^–^ content were observed in L1 and L2 under NaCl stress (Fig. 8D–F). These results indicate that MhCLC-c1 inhibits intracellular Cl^–^ accumulation under NaCl stress.

## Discussion

Excess NaCl in soil severely affects crop growth and yield. When crop suffer from NaCl stress, both Na^+^ and Cl^–^ cause cell damage and death of plants [7, 8], however, compared to Na^+^ toxicity, the Cl^–^ toxicity has not received enough attention [5, 16]. CLC are highly associated with Cl^–^ accumulation and confers NaCl stress tolerance in plants [17]. Apple roots, the organ that most direct and earliest identified soil NaCl stress, are susceptible to Cl^–^, and provided by rootstock in cultivated apple. Here, based the identification of apple *CLCs* gene family, we selected and isolated a *CLC-c1* homolog (*MhCLC-c1*) from the root of *M. hupehensis*, an excellent apple rootstock, and revealed that the *MhCLC-c1* alleviates the NaCl-induced cell death by inhibiting intracellular Cl^–^ accumulation.

To date, the *CLC* genes have been isolated in many species e.g., *Arabidopsis* [22], wheat [26], *Nicotiana tabacum* [27], soybean [31, 34], *Punica granatum* [29]. Here, we identified 9 *CLCs* from apple genome and divided them into two subclasses (Fig. 1). Among them, MdCLC-c1, MdCLC-d, and MdCLC-g were predicted that may be Cl^–^ antiporters or channels (Table 1), *MdCLC-a/b* and *MdCLC-c1, MdCLC-c2, MdCLC-f1,* and *MdCLC-g* had the higher expression level in root, especially *MdCLC-a/b* and *MdCLC-c1* (Fig. 2), and *MdCLC-c1* had the largest number of *cis-acting* elements associated with NaCl stress response namely 4 STRE and 1 TC-rich repeats (Fig. 3). Moreover, expression analysis showed the *MdCLC-c1* homolog *MhCLC-c1* expression in the roots of *M. hupehensis* held continued and rapid response during NaCl stress (Fig. 4). These data imply that *MhCLC-c1* may participate in NaCl stress response, but this need to be studied further.

*CLC-c* has been widely found that confers salt stress tolerance by regulating Cl^–^ accumulation. *Arabidopsis clc-c* mutants have lower Cl^–^ content in the guard cells and show hypersensitivity to Cl^–^ with a reduction in the shoot as well as root weight [24]. *GsCLC-c2* also confers Cl^–^/salt tolerance by regulating Cl^–^ transport from the roots to the shoots [28]. Here, since *MhCLC-c1* expression relative higher in roots and held continued and rapid response during NaCl stress, we isolated *MhCLC-c1* from *M. hupehensis* roots to study further (Fig. 5). Similar to CLC-c1 from apple and the other species, MhCLC-c1 also had two conserved CBS domain (Fig. 5A–C). Moreover, MhCLC-c1 was a PM-localized protein (Fig. 5D). However, it is reported that CLC-c was a vacuole intracellular membrane-localized protein in *Arabidopsis* [28], this difference may be due to the species difference. Furthermore, overexpression of *MhCLC-c1* decreased the apple calli and *Arabidopsis* sensitivity to NaCl stress, as well as the H_2_O_2_ content, O_2_^–^ content, REC, MDA content, and cell death of WT or Col-0 induced by NaCl stress, while suppression of *MhCLC-c1* significantly increased the H_2_O_2_ content, O_2_^–^ content, REC, MDA content, and cell death of WT (Figs. 6 and 7). Interestingly, overexpression of *MhCLC-c1* also increased LR number of *Arabidopsis* (Fig. 7A), which also confers *Arabidopsis* NaCl stress tolerance [35]. These results indicate that MhCLC-c1 negatively modulates apple sensitivity to NaCl stress and NaCl-induced cell death.

The inhibition of Cl^–^ accumulation confers plants NaCl stress tolerance and alleviates NaCl-induced cell death [7]. Here, overexpression of *MhCLC-c1* inhibited Cl^–^ accumulation of NaCl-stressed apple calli and *Arabidopsis*, while *MhCLC-c1* suppression increased Cl^–^ content in apple calli (Fig. 8A–F). As *MhCLC-c1* was repaid and continuous response to NaCl (Fig. 4), and negatively modulates apple sensitivity to NaCl stress and NaCl-induced cell death (Figs. 6 and 7), it is reasonable to believe that MhCLC-c1 negatively modulates NaCl-induced cell death by inhibiting intracellular Cl^–^ accumulation.

## Conclusion

In conclusion, *MhCLC-c1* expression was rapidly induced by NaCl, and the activated *MhCLC-c1* inhibits intracellular Cl^–^ overaccumulation caused by NaCl stress, thus the NaCl-induced ROS accumulation and cell death is alleviated (Fig. 8G). These results provide a new insight into the regulation of *MhCLC-c1* on NaCl-induced cell death. Overall, our findings confer the comprehensive and in-depth upstanding of the mechanism that plants resist salt stress, and might contribute to genetic improvement of salt tolerance in horticultural crops and the development and utilization of saline–alkali land.

## Methods

### Identification and characterization analysis of CLCs in apple

To identify the apple CLCs, 7 AtCLCs sequences were used as query sequences to screen *M. domestica* protein database using BLASTP (e-value ≤ 1e-5). Meanwhile, the conserved domain CBS (PF00571) of CLC was used as a query Hidden Markov Model (HMM) model to screen *M. domestica* protein database using hmmsearch 3.0. After combining the two results, and removing repetitive sequences, the rest sequences were submitted to SMART and NCBI-CDD to verify the conserved domain. The *Arabidopsis* AtCLCs sequences were downloaded from https://www.arabidopsis.org, and the the conserved domain CBS (PF00571) was downloaded from https://pfam.xfam.org/.

The theoretical isoelectric point (pI) and molecular weight (kDa) were determined using ExPASy (https://www.expasy.org) [36]. The subcellular localization were predicted using Wolf PSORT [37]. The conserved domain namely GxGxPE (I), GKxGPxxH (II), and PxxGxLF (III) were analyzed by MAST tool (http://meme-suite.org/tools/mast). The sequence alignment was carried out using DNAMAN. Genes and proteins annotated in apple (*Malus domestica*) were downloaded from https://www.rosaceae.org. The CLCs homologs from *Arabidopsis thaliana*, *Oryza sativa*, *Glycine max*, and *Nicotiana tabacum* were downloaded from https://www.ncbi.nlm.nih.gov/ and https://citrus.hzau.edu.cn/orange/. The phylogenetic analysis was conducted using MEGA 7.0 by the neighbor-joining method [38].

### Tissue expression profiles and *cis‑acting* elements prediction of *CLC* genes in apple

The GEO data of tissue expression profile (GSE42873) was downloaded from https://www.ncbi.nlm.nih.gov/geo/browse/. The RPKM values transformed by log2 of *MdCLCs* were used to generate a heatmap using Tbtools [39]. The 2 kb sequences from ATG upstream (promoters) were downloaded from http://www.phytozome.net, and the plantCARE was used to scanned the *cis‑acting* elements of *MdCLCs* promoters.

### Plant materials and growth conditions

*Malus hupehensis* Rehd. var. *pingyiensis Jiang* seedlings, ‘Orin’ apple calli, tobacco (*Nicotiana benthamiana*), and *Arabidopsis* (Columbia, Col-0) were used as materials in the study. *M. hupehensis* seeds were soaked in deionized water for 24 h, and then vernalized at 4℃ until budding. The budded seeds were grown in mixed substrate (3 turf: 1 perlite: 1 vermiculite) until they produced 1 or 2 leaves. The 1 or 2-leaf-old seedlings were transferred in ½ Hoagland’s nutrient solution until they produced six leaves. The apple calli was normally cultivated in Murashige and Skoog (MS) medium supplementing 0.4 mg/L N-(Phenylmethyl)-9H-purin-6-amine (6-BA), 1.5 mg/L 2-dichlorophenoxyacetic acid (2, 4-D), 3% (w/v) sucrose, and 0.8% (w/v) agar (pH 5.9) at 25 °C in the dark. Tobacco was normally cultivated in soil at 25 °C and 65% RH under a 16 h-light: 8-h dark photoperiod for 30 d. *Arabidopsis* seeds vernalized at 4 °C for 3 days, were seeded in ½ MS medium containing 3% (w/v) sucrose and 0.8% (w/v) agar (pH 5.9) and cultivated at 22 °C and 65% RH with a 16-h light: 8-h dark photoperiod.

### *M. hupehensis* seedlings NaCl treatments

Seedlings were transformed into ½ Hoagland’s nutrient solution contained 150 mM NaCl, and the roots were sampled at 0, 0.5, 1, 3, 7 d. All experiments were repeated three times. All samples were frozen quickly in liquid nitrogen and stored at –80 °C for expression analysis.

### Expression analysis

Total RNA extraction, RNA reverse transcription, and quantitative real-time PCR (qRT-PCR) were performed, as previously described [40]. All primers are listed in Supplemental Table 1. Nine replicates (3 technical replicates × 3 biological replicates) were performed per experiment. The heatmap was drawn using Tbtools [39].

### Isolation and subcellular localization of MhCLC-c1

The full-length cDNA of *MhCLC-c1* was amplified from *M. hupehensis* roots using Phanta® Max Super-Fidelity DNA Polymerase (Vazyme, Nanjing, China) and inserted into pBI121-GFP with the control of CaMV 35S promoter (35S:MhCLC-c1-GFP). To determine the subcellular localization of MhCLC-c1, pm-rk CD3-1007, a plasma membrane (PM)-located protein expression plasmid was used as a PM-marker [33]. The pBI121-GFP empty vector (35S:GFP) or 35S:MhCLC-c1-GFP were transiently co-transferred with pm-rk CD3-1007 into tobacco leaves using *Agrobacterium* GV3101 infiltration [41]. And the tobacco leaves after normally cultured for 3 d were observed using a laser confocal microscope (LSM880 Zeiss, Germany). Excitation wavelengths of 488 nm (GFP) and 561 nm (RFP) and receiving light wavelengths of 507 nm (GFP) and 610 nm (RFP) were used. The intensity curves of GFP and RFP was performed using ImageJ software.

### Generation and NaCl treatment of MhCLC-c1 transgenic apple calli and *Arabidopsis*

To determine the role of MhCLC-c1 in NaCl-induced cell death, the full-length cDNA of *MhCLC-c1* was inserted into pGWB405 to construct pGWB405-MhCLC-c1 fusion vector for *MhCLC-c1* overexpression using Gateway method [42]. Besides, a specific 200 bp sequence of its cDNA was reverse inserted into the pGWB405 vector for silence endogenous *MhCLC-c1* expression. The generation of *MhCLC-c1* transgenic apple calli was performed by *Agrobacterium* EHA105 infiltration as previously described [43]. Besides, the *Arabidopsis* overexpressing MhCLC-c1 was generated as our laboratory previous described [44].

For the apple calli, the 15-d-old wild-type (WT) apple calli, apple calli overexpressing MhCLC-c1 (OE), apple calli suppressing MhCLC-c1 (anti) with consistent growth were averagely divided into two groups, and the one group was plated on normal solid MS medium (Control), the other group was plated solid MS medium dissolved 200 mM (NaCl). After grown at 25℃ under dark conditions for 15 d, the phenotypes were photographed, and the cell damage, cell death and Cl^–^ content were measured. For the *Arabidopsis* plate experiments, the 4-d-old Col-0 and overexpressed *MhCLC-c1 Arabidopsis* (L1 and L2) were grown in ½ MS medium (Control) and ½ MS medium supplementing 100 mM NaCl (NaCl) grown for 5 d, then the roots phenotypes were photographed, the primary roots length and LR number were investigated, and the roots cell death was measured. For the *Arabidopsis* seedlings experiments on soil, the 10-d-old *Arabidopsis* seedlings grown in cultivated substrate (1 matrix: 1 vermiculite) were divided into two group with at least 10 plants in each group, and were watered using ½ Hoagland’s nutrient solution containing 200 mM NaCl once every two days. The phenotypes were photographed, and the roots cell death, H_2_O_2_ content and Cl^–^ content were detected after 10 d.

### Measurement of O_2_^–^ generation rate, H_2_O_2_ content, MDA content, and cell death

The O_2_^–^ generation rate, H_2_O_2_ content, and MDA content were measured using O_2_^–^ kit, H_2_O_2_ kit, and MDA kit according to their instructions, respectively (Suzhou Grace Biotechnolgy Co., Ltd, China). The REC was measured as described by Lutts et al. (1996) [45].

The cell death was determined using EB and FDA/PI staining. For the FDA/PI double staining of *Arabidopsis* seedlings, they were removed from MS medium or MS medium supplementing 100 mM NaCl and submerged in 2 ml FDA/PI work solution as described by pan et al. (2001) [46]. The LR were observed using a laser confocal microscope (LSM880 Zeiss, Germany), and excitation wavelength of 494 nm (FDA) and 535 nm (PI) and emission wavelength of 521 nm (FDA) and 615 nm (PI) were used. For EB staining of *Arabidopsis* roots on soil and apple calli, the *Arabidopsis* plants was slightly removed from cultivated substrate and its roots was gently shacked to dislodge the excess substrate, and the gently rinsed it off with moving water, then 0.1 g *Arabidopsis* or 0.3 g apple calli roots were randomly sampled and incubated with 0.25% (w/v) EB solution for 0.5 h, and rinsed in distilled water, while the apple calli also need to filtered with gauze. The stained apple calli was incubated with 5 mL 1% SDS solution for 24 h to obtain an extracting solution, and absorbance was measured at 600 nm.

### Measurement of Cl^–^ content

A Cl^–^-sensitive fluorescent probe MQAE (Beyotime, Shanghai, China) was used to indicate Cl^–^ content in apple calli and *Arabidopsis* roots cell and its fluorescence intensity gradually decreases with increasing Cl^–^ content. MQAE was prepared into 6 mM working solution with Krebs-HEPES buffer (20 mM HEPES, 128 mM NaCl, 2.5 mM KCl, 2.7 mM CaCl_2_, 1 mM MgCl_2_, 16 mM glucose, pH 6). The apple calli or *Arabidopsis* LR were submerged in 500 μL MAQE-HEPES buffer and incubated at 37 °C for 1 h. Then them were rinsed in distilled water and observed using a laser confocal microscope (LSM880 Zeiss, Germany). Excitation wavelength of 345 nm and emission wavelength of 455 nm was used. The fluorescence intensity was quantified by an ImageJ software. Quantitative analysis of Cl^–^ content was performed as our previous described [44].

### Statistical analysis

Data Processing System (DPS) were used to statistical the experimental data. Tukey's Method of one-way ANOVA was used to determine the significance of the differences among treatments (*p* < 0.05). Origin 2021 was used to draw the figures.

## Supplementary Information

**Additional file 1:** **Data S1.** The raw Ct value of 9 MhCLCs genes expression in response to NaCl analyzed by qRT-PCR. **Data S2.** The normalized Ct value of 9 MhCLCs genes expression in response to NaCl analyzed by qRT-PCR. **Data S3.** The fold_change of 9 MhCLCs genes expression in response to NaCl analyzed by qRT-PCR.

**Additional file 2: Figure S1.** The identification of transgenic apple calli and *Arabidopsis*. (A) DNA strip and expression analysis of WT and MhCLC-c1 transgenic apple callus; the primers are MhCLC-c1-F1 and MhCLC-c1-R1 in overexpression lines (OE); Anti-MhCLC-c1-F and Anti-MhCLC-c1-R in suppression lines (anti). The value for the WT was set to 1. (B) Expression analysis of MhCLC-c1 in Col-0 and transgenic *Arabidopsis*. The value for the Col-0 was set to 1. Bar represents mean ± SD and different letters above a bar represent a significant difference (*P* < 0.05). Each experiment was performed three biological repetition.

**Additional file 3:** **Table 1.** Primes used for vector construct and gene expression analysis. **Table 2.** The accession number of all genes.

## Data Availability

All data supporting the findings of this study are available within the paper and within its supplementary files.

## Abbreviations

Cl: Chloride
ROS: Reactive oxygen species
MDA: Malondialdehyde
CLC: Cl^–^ channel protein
CBS: Cystathionine β synthase
*M. hupehensis*: *Malus hupehensis* Rehd. var. *pingyiensis* Jiang
PM: Plasma membrane
WT: wild-type
FW: Fresh weight
EB: Evans blue
H_2_O_2_: Hydrogen peroxide
O_2_^–^: Superoxide anion
REC: Relative electric conductivity
LR: Lateral roots
MQAE: N-(Ethoxycarbonylmethyl)-6-Methoxyquinolinium Bromide
