# Supplementary material for: MhCLC-c1, a Cl channel c homolog from Malus hupehensis, alleviates NaCl-induced cell death by inhibiting intracellular Cl– accumulation
Source: BMC Plant Biol. 2023 Jun 8;23:306. doi: 10.1186/s12870-023-04270-3 (PMC10249150; doi:10.1186/s12870-023-04270-3)
Supplement: Supplementary file 2 — Additional file 2: Figure S1. The identification of transgenic apple calli and Arabidopsis. (A) DNA strip and expression analysis of WT and MhCLC-c1 transgenic apple callus; the primers are MhCLC-c1-F1 and MhCLC-c1-R1 in overexpression lines (OE); Anti-MhCLC-c1-F and Anti-MhCLC-c1-R in suppression lines (anti). The value for the WT was set to 1. (B) Expression analysis of MhCLC-c1 in Col-0 and transgenic Arabidopsis. The value for the Col-0 was set to 1. Bar represents mean ± SD and different letters above a bar represent a significant difference (P < 0.05). Each experiment was performed three biological repetition. [file 12870_2023_4270_MOESM2_ESM.docx]

Supplementary Figures


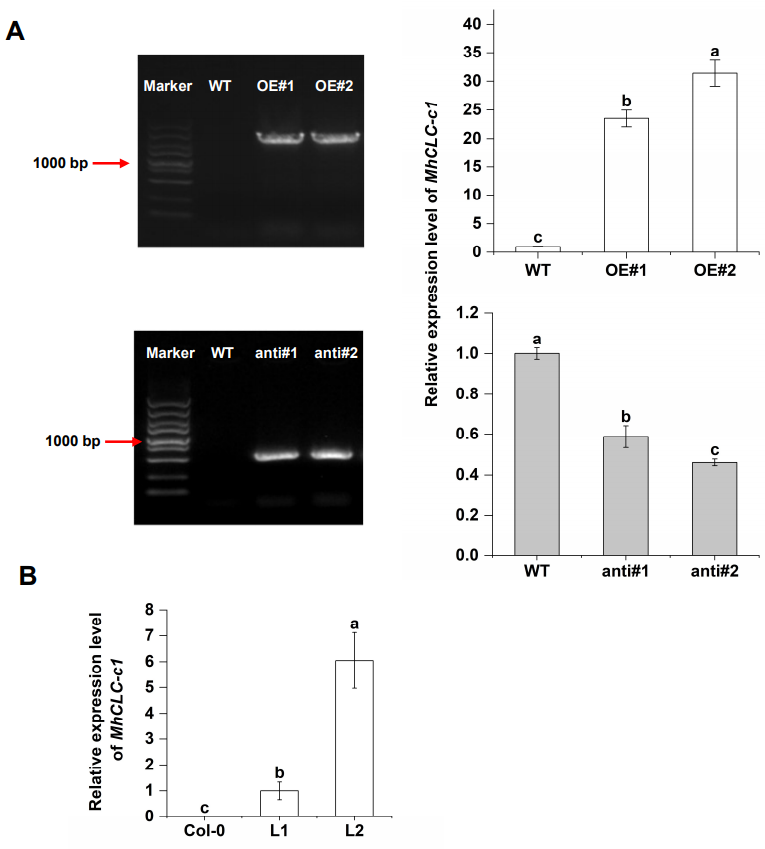


**Figure S1 The identification of transgenic apple calli and *Arabidopsis*.** (A) DNA strip and expression analysis of WT and MhCLC-c1 transgenic apple callus; the primers are MhCLC-c1-F1 and MhCLC-c1-R1 in overexpression lines (OE); Anti-MhCLC-c1-F and Anti-MhCLC-c1-R in suppression lines (anti). The value for the WT was set to 1. (B) Expression analysis of MhCLC-c1 in Col-0 and transgenic *Arabidopsis*. The value for the Col-0 was set to 1. Bar represents mean ± SD and different letters above a bar represent a significant difference (P < 0.05). Each experiment was performed three biological repetition.
