# Supplementary material for: MhCLC-c1, a Cl channel c homolog from Malus hupehensis, alleviates NaCl-induced cell death by inhibiting intracellular Cl– accumulation
Source: BMC Plant Biol. 2023 Jun 8;23:306. doi: 10.1186/s12870-023-04270-3 (PMC10249150; doi:10.1186/s12870-023-04270-3)
Supplement: Supplementary file 3 — Additional file 3: Table 1. Primes used for vector construct and gene expression analysis. Table 2. The accession number of all genes. [file 12870_2023_4270_MOESM3_ESM.docx]

**Supplementary Tables**

**Table 1 Primes used for vector construct and gene expression analysis**

| **Primer name** | **Primer sequence (5′- 3′)** | **Analysis** |
| --- | --- | --- |
| MhCLC-a/b F | TCCAATTCACAAGGAGACAGGT | qRT-PCR  qRT-PCR  qRT-PCR  qRT-PCR  qRT-PCR  qRT-PCR  qRT-PCR  qRT-PCR  qRT-PCR  qRT-PCR  qRT-PCR  qRT-PCR  qRT-PCR  qRT-PCR  qRT-PCR  qRT-PCR  qRT-PCR  qRT-PCR  qRT-PCR  qRT-PCR |
| MhCLC-a/b R | AGACGAGTGGGAGTATCTGGTA |  |
| MhCLC-c1 F | CTCCAGAGTTGTGCGGTCTT |  |
| MhCLC-c1 R | CCTTTCCTGACCCTGCCTTT |  |
| MhCLC-c2 F | TCTGATTACCTGCGGTGCTG |  |
| MhCLC-c2 R | AATGCCCTCCACAGAAGAGC |  |
| MhCLC-d F | CTGCTTCGTTGCAAAGCGAT |  |
| MhCLC-d R | AAGTTGAGAACCGGGAGTGC |  |
| MhCLC-e1 F | CACGTGCAGAATACCTGACATC |  |
| MhCLC-e1 R | TCAATCAGAGCGTCGACTGG |  |
| MhCLC-e2 F | AGTGCTTTCGTCGAGCTTGG |  |
| MhCLC-e2 R | TCTCAGCCATGAAGCAGAGT |  |
| MhCLC-f1 F | TAAGCGTGTCAGAGAGCCAC |  |
| MhCLC-f1 R | CCGGTGTAGTTTAAGAACAGGC |  |
| MhCLC-f2 F | CTGTAGATCACCACCACCATCA |  |
| MhCLC-f2 R | GAGAAGCAAAGCCCATTCGG |  |
| MhCLC-g F | GACACGTCCGGTATCTCGTT |  |
| MhCLC-g R | AAACTACTGCCCTTCCGGTC |  |
| Mhactin F | TAAGGCTGGATTTGCTGGAG |  |
| Mhactin R | GCATCTTTCTGACCCATTCC |  |
| At18S rRNA F | TTGCGTTTGAGAGGATGTGG | qRT-PCR |
| At18S rRNA R | TCCATAAGTCGGGGTTTGTTG | qRT-PCR |
| MhCLC-c1-F1 | ggggacaagtttgtacaaaaaagcaggcttc  ATGATGAATGAAGAAAATGGAAGCA | Vector construction for genetic transform |
| MhCLC-c1-R1 | ggggaccactttgtacaagaaagctgggtt  TGACTTATGAGCCTTTATATGCGG | Vector construction for genetic transform |
| Anti-MhCLC-c1-F | CATATGCCCGTCGACCCCGGG  GTCAGACATGAGAGCTGTGGTCAA | Vector construction for genetic transform |
| Anti-MhCLC-c1-R | TCAGAATTCGGATCCGGTACC  ATGATGAATGAAGAAAATGGAAGCA | Vector construction for genetic transform |
| MhCLC-c1-F2 | ggacaagggtctagacccggg ATGATGAATGAAGAAAATGGAAGCA | Vector construction for Subcellular location |
| MhCLC-c1-R2 | tctatcgatcaatcaggatcc GTCAGACATGAGAGCTGTGGTCAA | Vector construction for Subcellular location |

**Table 2 The accession number of all genes**

| **Species** | **Gene name** | **Accession number** |
| --- | --- | --- |
| *Malus hupehensis* | *MhCLC-c1* | MZ665463 |
| *Malus domastica* | *MdCLC-a/b* | MDP0000319946 |
| *Malus domastica* | *MdCLC-c1* | MDP0000239069 |
| *Malus domastica* | *MdCLC-c2* | MDP0000403033 |
| *Malus domastica* | *MdCLC-d* | MDP0000291244 |
| *Malus domastica* | *MdCLC-e1* | MDP0000132079 |
| *Malus domastica* | *MdCLC-e2* | MDP0000159684 |
| *Malus domastica* | *MdCLC-f1* | MDP0000240183 |
| *Malus domastica* | *MdCLC-f2* | MDP0000302319 |
| *Malus domastica* | *MdCLC-g* | MDP0000314369 |
| *Malus domestica* | *MdActin* | LOC103418811 |
| *Arabidopsis thaliana* | *AtCLC-a* | AT5G40890 |
| *Arabidopsis thaliana* | *AtCLC-b* | AT3G27170 |
| *Arabidopsis thaliana* | *AtCLC-c* | AT5G49890 |
| *Arabidopsis thaliana* | *AtCLC-d* | AT5G26240 |
| *Arabidopsis thaliana* | *AtCLC-e* | AT4G35440 |
| *Arabidopsis thaliana* | *AtCLC-f* | AT1G55620 |
| *Arabidopsis thaliana* | *AtCLC-g* | AT5G33280 |
| *Arabidopsis thaliana* | *At18S rRNA* | X16077 |
| *Oryza sativa Japonica Group* | *OsCLC-a* | XP_015620662.1 |
| *Oryza sativa Japonica Group* | *OsCLC-c1* | XP_015633162.1 |
| *Oryza sativa Japonica Group* | *OsCLC-c2* | XP_015626588.1 |
| *Oryza sativa Japonica Group* | *OsCLC-d* | AAO19370.1 |
| *Oryza sativa Japonica Group* | *OsCLC-e* | XP_015622009.1 |
| *Oryza sativa Japonica Group* | *OsCLC-g1* | XP_015650515.1 |
| *Oryza sativa Japonica Group* | *OsCLC-g2* | XP_015636607.1 |
| *Nicotiana tabacum* | *NtCLC-b1* | XP_016442033.1 |
| *Nicotiana tabacum* | *NtCLC-b2* | NP_001312163.1 |
| *Nicotiana tabacum* | *NtCLC-c1* | XP_019242943.1 |
| *Nicotiana tabacum* | *NtCLC-c2* | NP_001312418.1 |
| *Nicotiana tabacum* | *NtCLC-c3* | XP_009618449.1 |
| *Nicotiana tabacum* | *NtCLC-c4* | XP_016485010.1 |
| *Nicotiana tabacum* | *NtCLC-d* | XP_016512457.1 |
| *Nicotiana tabacum* | *NtCLC-e* | XP_016461327.1 |
| *Nicotiana tabacum* | *NtCLC-f1* | XP_016477395.1 |
| *Nicotiana tabacum* | *NtCLC-f2* | XP_016433983.1 |
| *Nicotiana tabacum* | *NtCLC-f3* | XP_016445164.1 |
| *Nicotiana tabacum* | *NtCLC-g1* | XP_016433616.1 |
| *Nicotiana tabacum* | *NtCLC-g2* | XP_016468446.1 |
| *Glycine max* | *GmCLC-d* | XP_003517583.1 |
| *Glycine max* | *GmCLC-1* | AAY43007.1 |
| *Nicotiana sylvestris* | *NsCLC-c* | XP_009768532.1 |
